# The use of DAPI fluorescence lifetime imaging for investigating chromatin condensation in human chromosomes

**DOI:** 10.1038/srep31417

**Published:** 2016-08-16

**Authors:** Ana Katrina Estandarte, Stanley Botchway, Christophe Lynch, Mohammed Yusuf, Ian Robinson

**Affiliations:** 1 London Centre for Nanotechnology, University College London, London, WC1H 0AH, UK; 2 Research Complex at Harwell, Rutherford Appleton Laboratory, Oxon, OX11 0FA, UK; 3 Central Laser Facility, Science and Technology Facilities Council, Rutherford Appleton Laboratory, Oxon, OX11 0QX, UK

## Abstract

Chromatin undergoes dramatic condensation and decondensation as cells transition between the different phases of the cell cycle. The organization of chromatin in chromosomes is still one of the key challenges in structural biology. Fluorescence lifetime imaging (FLIM), a technique which utilizes a fluorophore’s fluorescence lifetime to probe changes in its environment, was used to investigate variations in chromatin compaction in fixed human chromosomes. Fixed human metaphase and interphase chromosomes were labeled with the DNA minor groove binder, DAPI, followed by measurement and imaging of the fluorescence lifetime using multiphoton excitation. DAPI lifetime variations in metaphase chromosome spreads allowed mapping of the differentially compacted regions of chromatin along the length of the chromosomes. The heteromorphic regions of chromosomes 1, 9, 15, 16, and Y, which consist of highly condensed constitutive heterochromatin, showed statistically significant shorter DAPI lifetime values than the rest of the chromosomes. Differences in the DAPI lifetimes for the heteromorphic regions suggest differences in the structures of these regions. DAPI lifetime variations across interphase nuclei showed variation in chromatin compaction in interphase and the formation of chromosome territories. The successful probing of differences in chromatin compaction suggests that FLIM has enormous potential for application in structural and diagnostic studies.

Chromosomes are composed of chromatin, a complex of DNA and proteins, which are arranged into elementary structural units of nucleosomes. Each nucleosome consists of 147 base pairs of DNA wrapped around a core of eight histone proteins^1^. These nucleosomes are arranged into 11-nm fibers having a “beads-on-a-string” appearance^2^. As the cell cycle proceeds from interphase to metaphase, the chromatin undergoes further condensation by the addition of a protein scaffold thus forming higher order structures until the chromosomes reach the metaphase stage of mitosis, their most compact state^3^. The organization of chromatin into these higher-order structures and the factors that play a role in the condensation process remain a subject of debate and represent one of the key challenges in structural biology.

Chromatin has historically been categorized into two structural states: heterochromatin and euchromatin^4^. Heterochromatin has been described as the chromatin fraction that is more compact and remains condensed throughout the cell cycle except during its replication. It is thought to be inactive in transcription, has a low gene density, and is replicated late in the S-phase^5^,^6^. It can be further classified into constitutive heterochromatin, which is composed of repetitive sequences of DNA known as satellite repeats and is associated mostly with pericentromeric and telomeric regions of the chromosome, and facultative heterochromatin, which can interconvert between heterochromatic and euchromatic states when triggered by several factors such as acetylation and methylation^7^,^8^. While euchromatin has been described as the chromatin fraction that is relatively decompacted and decondenses during interphase, it is active in transcription, more likely to contain genes, and is replicated early in S-phase^5^,^6^.

Visualization of distinct subchromosomal regions can be achieved through so-called banding, a characteristic striped appearance that results from the differential staining along the length of a chromosome. Bands also allow the identification of individual chromosomes and the presence of possible abnormalities therein such as deletions, insertions, and translocations and are used in cytogenetic clinical laboratories. The common banding patterns observed are G-, R-, and C- banding patterns^9^. Usually, the contrast between the bands is based on the relative intensity of the stain.

A vital characteristic of a fluorophore apart from excitation, emission wavelength, and intensity is its fluorescence excited state lifetime (τ), which can be defined as the average time a fluorophore stays in the electronic excited state. There are different pathways of relaxation (e.g., internal conversion, dynamic quenching, energy transfer) for a fluorophore in the excited state aside from fluorescence which can all compete kinetically with fluorescence and thereby affect the fluorescence rate^10^. The rates of these relaxation pathways are sensitive to many factors such as proximity of the fluorophore to other fluorophores and quenching species as well as the dielectric constant of the medium. Hence, fluorescence lifetime can change markedly depending on a fluorophore’s environment.

Fluorescence lifetime imaging microscopy (FLIM) is a technique that maps the spatial arrangement of lifetime and can act as a probe to changes in the fluorophore’s environment. A lifetime image is acquired wherein the fluorescence lifetime is measured, with temporal resolution of nanoseconds or less, at every pixel of the image. FLIM has several applications across both physical and life sciences. It is widely used for investigations at Förster resonance energy transfer (FRET) lengths, typically from 1–10 nm, between a fluorophore and another molecule to probe distances and study interactions between molecules (e.g., homo- and hetero-dimerizations and protein-protein interactions^11^,^12^). A major advantage of FLIM is that it is not explicitly dependent upon molecular concentrations of the fluorophore unlike intensity-based fluorescence imaging^10^. This is useful because the fluorophore concentration across a sample may be far from uniform. However, at relatively high (mM) fluorophore concentrations, self-quenching of the fluorophore may occur which can then affect the fluorescence lifetime of the fluorophore^13^,^14^.

Little is known about the sensitivity of fluorescence lifetime to changes in chromosome environment. Llères *et al*.^15^ used FLIM to measure FRET between histone molecules tagged with either GFP or mCherry in interphase chromosomes inside the nucleus of HeLa cells. Different FRET efficiency populations were observed across the interphase nucleus, reflecting regions with different levels of chromatin compaction. The frequencies of the different FRET efficiency populations were observed to change at different stages of mitosis, from metaphase to telophase. The study, however, did not investigate in depth and at high resolution the variations in FRET efficiency along the length of individual chromosomes.

In this work, FLIM is used to investigate the different chromosome substructures at the nanometer length scales by the variations in the DAPI (4′-6-diamidino-2-phenylindole) excited state lifetime loaded into human metaphase chromosomes and interphase nuclei. DAPI is a nucleic-acid specific fluorophore that is widely used in chromosome staining because of its high quantum yield (ϕ_f_ = 0.92) when bound to DNA^16^. Quantum yield is a parameter which shows the emission efficiency of a given fluorophore and is equivalent to the ratio of number of photons emitted to the number of photons absorbed. The ϕ_f_ of unbound DAPI (0.04) is several orders of magnitude less than in the presence of DNA. DAPI is known to bind preferentially to AT base pairs in the minor groove of the DNA^17^,^18^. However, other binding modes of DAPI are also observed at high DAPI loadings such as external binding through electrostatic interactions with the phosphate groups of the DNA and intercalation between GC base pairs^19^,^20^,^21^,^22^.

Here we observed variations in the DAPI excited state lifetime along the length of the metaphase chromosomes and across the interphase nuclei that correlate with differentially compacted regions of chromatin. The pericentromeric regions of chromosomes 1, 9, and 16, short arm of chromosome 15, and distal region of chromosome Y in the metaphase spreads showed significantly shorter lifetime values (τ_1,16,Y_ = 2.57 ± 0.06 ns, τ_9a,15_ = 2.41 ± 0.06 ns, and τ_9b_ = 2.21 ± 0.05 ns) as compared with the rest of the chromosomes (τ = 2.80 ± 0.09 ns). What we now provide is a detailed quantitative analysis of all the chromosomes in a mammalian cell which isn’t present in previous reports.

## Materials and Methods

### DNA Preparation, Cell Culture, and Chromosome Extraction

0.001 mg/mL of calf thymus and micrococcus luteus DNA (Sigma Aldrich, UK) in deionized water were prepared and stained with 4 μM DAPI (Invitrogen, UK). Drops from these solutions were then placed on coverslips for fluorescence lifetime measurements.

Chromosomes were prepared from suspension B-lymphocyte cells (GM18507, International HapMap Project, Yoruba male). This cell line was at passage four and prepared according to a previously published protocol^23^. The cells were cultured in a suspension of RPMI-1640 medium (Sigma Aldrich, UK) supplemented with 20% foetal bovine serum (FBS) (Sigma Aldrich, UK) and 1% L-glutamine (Sigma Aldrich, UK) at 37 °C in a 5% CO_2_ incubator. For synchronization of the cell cycle, the cells were treated with thymidine (Sigma Aldrich, UK) at a final concentration of 0.3 mg ml^−1^ for 17 hours. To obtain chromosomes at the mitotic stage, colcemid (Invitrogen, UK) was added to the cells at a final concentration of 0.2 μg ml^−1^. The cells were then left for 16 hours before harvesting. This was followed by hypotonic treatment of the cells with 0.075 M potassium chloride for five minutes. Following the hypotonic treatment, the samples were fixed in three changes of 3:1 methanol:acetic acid solution.

Chromosomes were also prepared from adherent cervical cancer cells (HeLa) and normal lung fibroblast cells from a healthy female (CCD37LU). The cells were cultured in Dulbecco’s Modified Eagle’s medium (DMEM) (Sigma Aldrich, UK) supplemented with 10% FBS and 1% L-glutamine at 37 °C in a 5% CO_2_ incubator. The same procedures above were followed for the synchronization of the cells. After the colcemid treatment, trypsin was added to the cells to detach them from the substrate. The same hypotonic treatment as above was then followed and the resulting samples were fixed in three changes of 3:1 methanol:acetic acid solution.

Even though synchronization of cells was performed during the chromosome preparation procedures above, not all chromosomes obtained had reached the metaphase stage. The final chromosome samples fixed in methanol:acetic acid solution still contained some interphase chromosomes.

To prepare the chromosome samples for FLIM, the chromosomes were spread on glass slides using the hanging drop method and stained with either 4 μM DAPI or 4 μM Hoechst 33258 for five minutes. The glass slides were washed in a 1x phosphate buffer saline (PBS) solution (Sigma Aldrich, UK) for 5 minutes. The samples were covered with a coverslip, with deionized water as the mounting medium.

### Chromosome Identification using mFISH

In order to identify the chromosomes in the samples measured with FLIM, multiplex fluorescence *in-situ* hybridization (mFISH) was performed using a 24XCyte mFISH probe kit (MetaSystems, Germany) following the recommended procedure of the manufacturer and according to a previously published protocol^24^. The kit consists of 24 painting probes specific for the 24 different human chromosomes. Each probe is labeled with up to five different fluorophores in a combinatorial labeling format to provide 24 distinct colors. The hybridization of the probe with the DNA sites was visualized by fluorescence microscopy using a Z2 Zeiss fluorescence microscope. mFISH images were then analyzed using the ISIS software from MetaSystems.

### Fluorescence Lifetime Measurements of the DNA and Chromosome Samples

Multiphoton fluorescence lifetime measurements of the chromosome samples were performed at the Central Laser Facility (CLF) located at the Research Complex at Harwell, UK. The custom set up has been previously described^25^. Briefly, a femtosecond Ti-sapphire laser operating at 76 MHz, 200 fs pulse lengths was tuned to a wavelength of 760 nm for two-photon excitation of DAPI and Hoechst 33258 fluorescence. The use of multiphoton excitation here allowed for instrument response function (IRF) of less than 45 ps together with a micro-channel plate (MCP) photomultiplier tube (R3809U, Hamamatsu) as the detector. This was necessary to detect small variations in the lifetime measurements without the need for significant deconvolution of the data from the IRF. The samples were placed on a Nikon TE2000 sample stage attached to a modified Nikon EC2 confocal microscope and were raster-scanned with the laser through a x60 NA 1.2 water immersion objective. The fluorescence from the sample was collected by the same objective but bypassing the confocal optics and detected by a fast MCP-photomultiplier tube detector through a short-pass filter (BG39, Comar, UK). A time-correlated single photon counting (TCSPC) FLIM module (SPCM-830) from Becker & Hickl was used to record the arrival time of the photons at each x, y scan position in a time-tagging, first-in first-out mode.

The same set-up was used for the measurement of the DNA samples. However, since the samples were in solution, raster scanning was not performed and a single point collection mode was selected. One DAPI fluorescence decay curve was obtained for every DNA sample measured.

### Data Analysis of Lifetime Images

The acquired fluorescence lifetime images (up to 512 × 512 pixels) were analyzed using the SPCImage software (version 4.0.6). Each pixel of the acquired images contains a fluorescence decay function of the DAPI lifetime. The measured decay function (Eq. 1) is a convolution of the true decay function with the IRF. The software uses a model function (Eq. 2), which can be a single or multi-exponential function, to define the fluorescence decay function f(t) and convolutes it with the IRF. The result is compared with the measured decay function. The parameters of the model function are varied until the best fit with the measured decay function is obtained. The lifetime is then extracted from the fitted function thus resulting in an image with a lifetime value per image pixel.


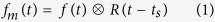



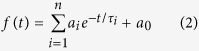


where f_m_(t) is the measured fluorescence decay function, f(t) is the true fluorescence decay function, R is the instrument response function, t_s_ is the time shift between the response function and the fluorescence, a_i_ is the relative amplitude of the exponential component i, τ_i_ is the lifetime of the exponential component i, and a_0_ is a constant offset-correction.

## Results

### Excited State Characteristics of Free DAPI and DAPI Bound to DNA

The excited state lifetime of DAPI is well known^26^, so we measured the lifetime of unbound DAPI in PBS solution (pH 7.2) to determine both the sensitivity of the system and the accuracy of lifetime measurements. The fluorescence decay showed a double exponential character: fluorescence lifetime components, τ, and intensity coefficients, a, are reported in Table 1. The value of the long lifetime component agrees well with literature while the value of the short lifetime component is higher than that described in the literature at pH 7 where the value range is 0.19–0.24 ns^26^.

We next measured lifetimes of DAPI bound to DNA containing different amounts of AT and GC bases to determine if DNA base composition has any effect on the DAPI lifetime. Calf thymus (CT) and micrococcus luteus (ML) DNA were used for this part of the study because of their different base pair compositions (42% GC for CT DNA and 72% GC for ML DNA). Three solutions each of CT and ML DNA were measured. The DAPI lifetimes for each type of DNA exhibited two components (Table 1). The short lifetime component of DAPI bound to ML DNA is slightly higher than that of the CT DNA (130 ps difference). There is no significant difference observed between the long lifetime components.

### FLIM of DAPI Bound to Fixed Metaphase Chromosomes from B-Lymphocytes

**Figure 1:**
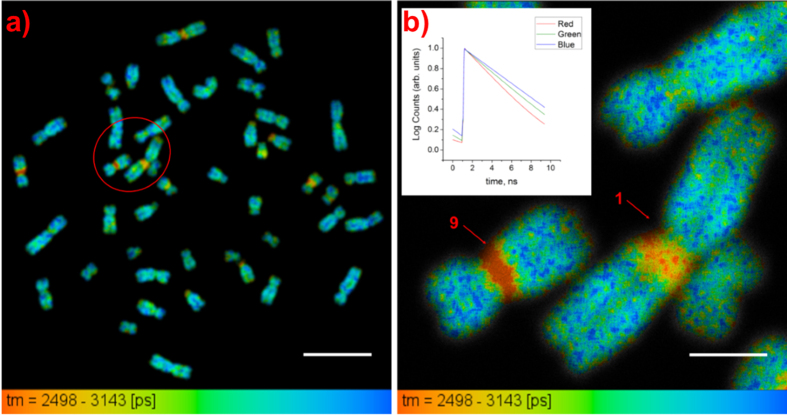
Variations in DAPI lifetime along the length of the chromosomes. Lifetime images of (**a**) a chromosome spread (scale bar = 10 μm) and (**b**) an expanded image of the enclosed region in Fig. 1a taken at a higher pixel resolution showing chromosomes 1 and 9 (scale bar = 2 μm). Figure 1b inset: normalized DAPI fluorescence decay curves at selected pixels from the red, green, and blue regions in chromosome 9 in Fig. 1b. The range of the lifetime color scale runs from 2.50 ns (red) to 3.14 ns (blue), as shown.

Figure 1a shows a lifetime image of a typical “spread” of human metaphase chromosomes fixed in 3:1 methanol:acetic acid (see Methods for definition of chromosome spread). An expanded image of the enclosed region in the figure taken at a higher pixel resolution is shown in Fig. 1b. False color is used to represent the lifetime value at each image pixel.

Both figures show that there is a variation in the lifetime of DAPI along the length of the chromosomes. The DAPI fluorescence decay curves with normalized intensities are also presented in Fig. 1b to complement the lifetime images and show that the fluorescence of the DAPI molecules in the red regions has a faster decay than that in the blue regions. The mean lifetime ± standard deviation (SD) of DAPI for the measured chromosome spread in Fig. 1a was determined to be 2.91 ± 0.12 ns; the fluorescence decay of DAPI was found to have single exponential characteristic.

### Identification of Heteromorphic Regions in Chromosomes

**Figure 2:**
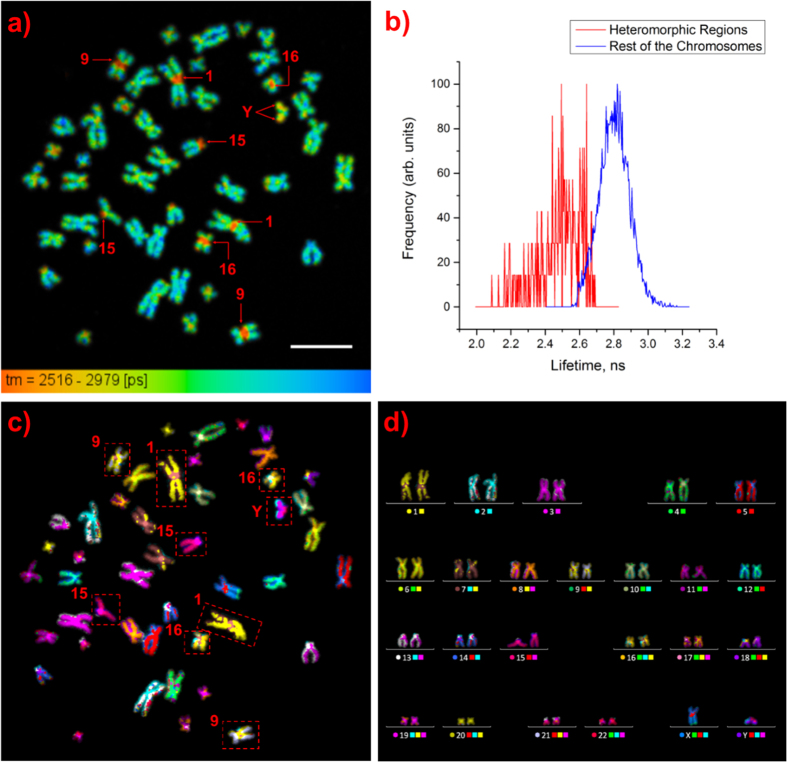
Identification of heteromorphic regions in chromosomes. (**a**) Lifetime image of a chromosome spread with arrows showing the heteromorphic regions (scale bar = 10 μm). (**b**) Normalized lifetime distribution curves for the heteromorphic regions and the rest of the chromosomes showing shorter DAPI lifetimes for the heteromorphic regions than for the rest of the chromosomes. (**c**) mFISH image of the measured chromosome spread. (**d**) Karyotyping of the chromosomes in Fig. 2a,c based on color.

Twelve chromosome spreads (GM18507 cells) with 46 chromosomes each were measured from three slides. Figure 2a shows a lifetime image of one of the measured spreads. In each of the measured spreads we found that certain chromosomes have specific regions along their lengths that have significantly shorter lifetimes than the rest of the chromosome, mostly in close proximity to the centromeres. These regions appear red in lifetime images because of the color scale used; we shall refer to them as short lifetime regions.

The lifetime distribution curves with normalized frequencies for the short lifetime regions and the rest of the chromosomes in Fig. 2a are shown in Fig. 2b. The mean lifetime ± SD of DAPI molecules in the short lifetime regions in Fig. 2a was determined to be 2.48 ± 0.13 ns while that for the molecules in the rest of the chromosomes in the spread was determined to be 2.80 ± 0.09 ns. The standard deviations represent how varied the lifetime values are within the short lifetime regions and the rest of the chromosomes for the measured spread.

We suspected that the short lifetime regions corresponded to heteromorphic regions in chromosomes. Such regions display variations in morphology at specific locations whilst not affecting phenotype, and are often heterochromatic in nature. Known morphological variations of heteromorphic regions have been associated with longevity and infertility, and so are of great interest in genetics studies. Heteromorphic regions are known to occur on chromosomes 1, 9, 15, 16 and the Y chromosome^27^.

It was decided to perform mFISH experiments (see Methods) on all of the measured spreads in order to identify the chromosomes containing the short lifetime regions. The mFISH image and karyotype of the spread in Fig. 2a are shown in Fig. 2c,d, respectively. The regions showing shorter lifetimes, as compared with the rest of the chromosomes, in all of the measured spreads were identified to be the pericentromeric regions of chromosomes 1, 9, and 16, the short arm of chromosome 15, and the distal region of chromosome Y; these regions are known to be heteromorphic regions, confirming our hypothesis.

Table 2 shows the mean lifetime value of DAPI for each of the heteromorphic regions in Fig. 2a. As each autosome appears twice, the mean lifetimes and standard deviations for the heteromorphic regions obtained for autosomes with the same chromosome number were averaged and pooled, respectively.

It can be observed from the table that the measured lifetimes for the heteromorphic regions of chromosomes 1, 16, and Y are similar and are significantly longer than those of chromosomes 9 and 15. The heteromorphic region of chromosome 9 can be described by two lifetimes so that region 9a has a similar value to that of chromosome 15 while region 9b has a shorter lifetime than that of region 9a.

The other measured spreads (see Tables S1–S11 in the Supplementary Materials) show a similar trend of the heteromorphic regions of chromosomes 9 and 15 having shorter lifetimes than those of chromosomes 1, 16, and Y.

### Trends in DAPI Lifetime with Chromosome Area

Chromosomes are known to become progressively more compact as they approach the metaphase stage of the cell cycle; Colcemid was used for this study to block the cells at this stage before harvesting. However, the cells in the synchronized population are not all expected to be exactly in the same stage of the condensation upon harvest. This variation in the compaction is reflected in the variation in the relative area of a particular chromosome from one chromosome spread to another on the same slide, with the more compact chromosomes having smaller areas. We anticipated that different states of compaction might have an effect on the fluorescence lifetimes observed. To measure the effect of chromatin compaction on lifetimes, we correlated the mean DAPI lifetimes for various examples of chromosome 1’s (GM18507 cells) from one slide and their heteromorphic regions with the measured chromosome area. As described earlier, the lifetime values for chromosome pairs were averaged, pixels assigned a color according to lifetime value and short lifetime regions (red regions) were taken to be heteromorphic in nature. Segmentation and analysis of the heteromorphic regions and calculation of the chromosome areas were carried out using the Avizo software.

**Figure 3:**
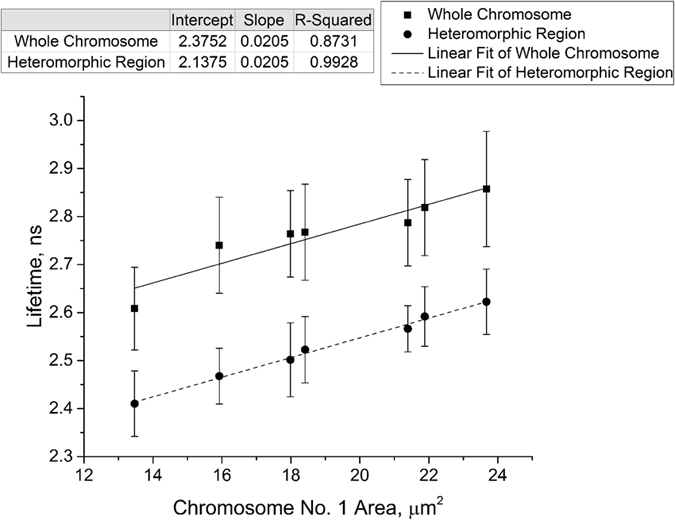
Mean fluorescence lifetime of DAPI for various chromosome 1’s and their heteromorphic regions plotted against the area of the chromosomes. The error bars represent the standard deviation. Thus, DAPI is sensitive to both general chromosome length compactions as well as localized sub-chromosome condensation.

The graph in Fig. 3 shows that the mean lifetimes of DAPI for chromosome 1 and its heteromorphic region decrease strongly with decreasing chromosome 1 area. To the extent to which the area of a chromosome in a spread represents packing density, the observed trend suggests a linear dependence on density, representing the degree of condensation. A similar trend is also observed for chromosome spreads on another slide (see Figure S1).

### Effect of DAPI Concentration on Lifetime

The mean DAPI lifetimes for various chromosomes 1’s (GM18507 cells) stained with different DAPI concentrations were measured to determine the effect of DAPI concentration on the lifetime. Twelve chromosome 1’s, with approximately the same area, from the same slide were measured for each concentration.

It can be observed from Table 3 that the total intensity of chromosome 1 increased as the concentration of DAPI was increased. However, there was no significant variation observed in the DAPI lifetime between the different concentrations.

### FLIM of Hoechst 33258 Bound to Fixed Metaphase Chromosomes

In order to confirm that the lifetime variations we have observed along the length of the chromosomes is a function of chromatin structure and not due to any effects specific to DAPI, we carried out FLIM using another minor-groove binding dye. Metaphase chromosomes (GM18507 cells) fixed in 3:1 methanol:acetic acid were stained with Hoechst 33258.

**Figure 4:**
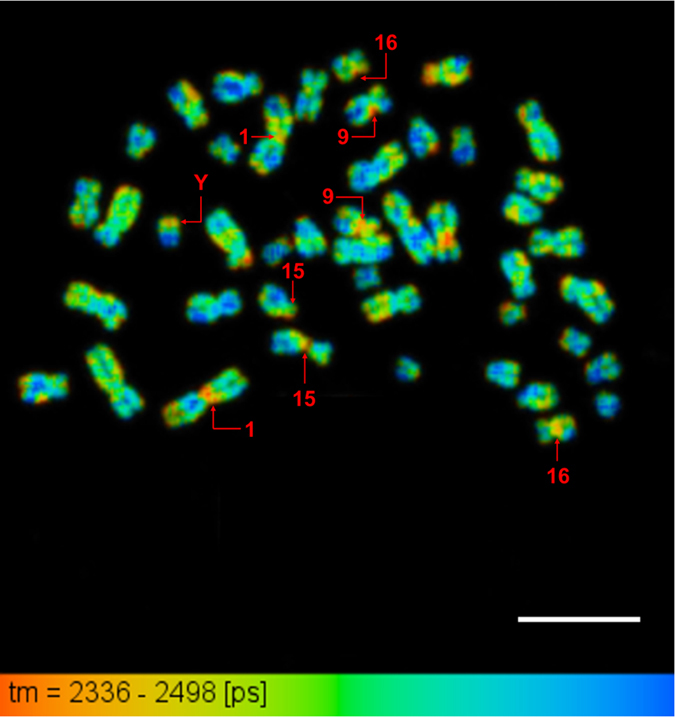
Lifetime image of a chromosome spread stained with Hoechst 33258 with arrows showing the heteromorphic regions (scale bar = 10 μm).

Figure 4 shows the lifetime image of the measured chromosome spread. The fluorescence decay of Hoechst 33258 bound to the chromosomes was found to have single exponential characteristics, similar to that of DAPI. The mean lifetime ± SD of Hoechst 33258 for the measured spread was determined to be 2.42 ± 0.05 ns. The smaller standard deviation, as compared with that of the DAPI-stained chromosomes (0.12 ns), shows that the variation in the Hoechst 33258 lifetime across the spread is not as high as that observed for the DAPI-stained chromosomes. The lifetime image shows that the heteromorphic regions of chromosomes 1, 9, 15, 16, and Y displayed significantly shorter lifetime values than the rest of the chromosomes (see Figure S2 for the mFISH image and karyotype), similar to that observed for the DAPI-stained chromosomes. The mean lifetime ± SD of Hoechst 33258 molecules in the heteromorphic regions was determined to be 2.36 ± 0.03 ns while that for the molecules in the rest of the chromosomes in the spread was determined to be 2.43 ± 0.04 ns. The mean Hoechst 33258 lifetime values and standard deviations for each of the heteromorphic regions of chromosomes 1, 9, 15, 16, and Y, as compared with the rest of the chromosome, are presented in Table S12.

This additional experiment using Hoechst confirms that the lifetime variations observed along the length of the chromosomes are caused by the local chromatin structure and not the fluorophore used.

### FLIM of DAPI Bound to Fixed Metaphase Chromosomes from HeLa Cells

FLIM measurements on DAPI-stained metaphase chromosomes obtained from HeLa cells fixed in 3:1 methanol:acetic acid were also performed to verify that the variations in the DAPI lifetime observed along the length of the chromosomes are not just cell line specific but are due to the general chromatin structure.

**Figure 5:**
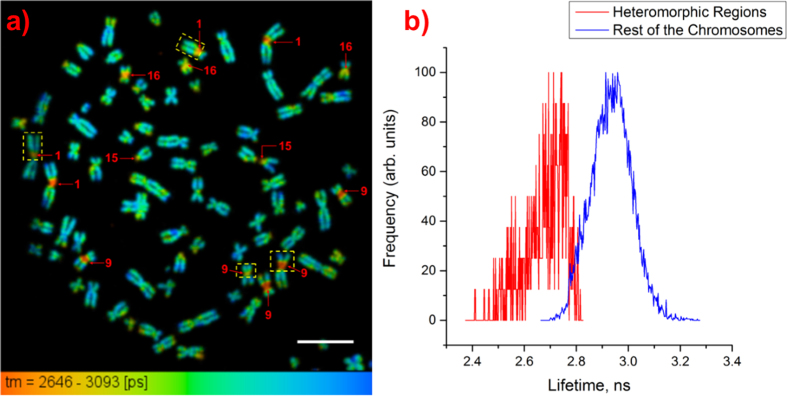
Identification of heteromorphic regions in chromosomes obtained from HeLa cells. (**a**) Lifetime image of a chromosome spread with arrows showing the heteromorphic regions. The chromosomes enclosed in a yellow dashed square are the abnormal chromosomes (scale bar = 10 μm). (**b**) Normalized lifetime distribution curves for the heteromorphic regions and the rest of the chromosomes showing shorter DAPI lifetimes for the heteromorphic regions than for the rest of the chromosomes.

Figure 5a shows the measured chromosome spread. The mean lifetime ± SD of DAPI for the measured spread was determined to be 2.93 ± 0.09 ns. Shorter DAPI lifetimes, as compared with the rest of the chromosomes, are observed at the heteromorphic regions of chromosomes 1, 9, 15, 16, and Y (see Figure S3 for the mFISH image and karyotype), similar to that observed for the GM18507 cells. Four abnormal chromosomes consisting a part of either chromosome 1 or 9 also exhibited the shorter DAPI lifetimes in their pericentromeric regions suggesting that they contain the heteromorphic region of chromosome 1 or 9. The lifetime distribution curves with normalized frequencies for the short lifetime regions and the rest of the chromosomes are shown in Fig. 5b. The mean lifetime of the DAPI molecules in the short lifetime regions was determined to be 2.68 ± 0.08 ns while that for the molecules in the rest of the chromosomes in the spread was determined to be 2.93 ± 0.08 ns.

### FLIM of DAPI Bound to Interphase Chromosomes within Fixed Nuclei

**Figure 6:**
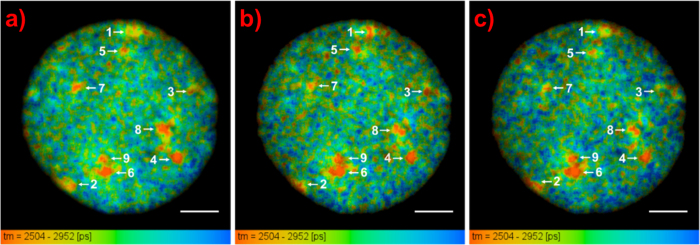
Selected focal planes from the z-stack of the lifetime images of interphase nucleus from GM18507 cells at (**a**) −0.50 μm, (**b**) 0 μm, and (**c**) +0.50 μm focus (scale bars = 5 μm, see Figure S5 for all the focal planes in the z-stack).

The lifetime of DAPI bound to interphase chromosomes within a 3:1 methanol:acetic acid fixed nucleus from the GM18507 lymphocyte cell line was also imaged, as shown in Fig. 6. A z-stack of the nucleus was taken using a multiphoton excitation (at 760 nm) confocal microscope. Fig. 6a,c show images obtained at −0.50 μm and +0.50 μm, respectively, from the original focal plane (Fig. 6b).

A single exponential character was observed for the DAPI fluorescence decay. The lifetime images of the measured nucleus show that there is a strong variation in the DAPI lifetime across the interphase nucleus, where localized regions of short lifetimes can be observed (indicated by arrows in Fig. 6). Even though the fixed nucleus is somewhat collapsed compared to its native spherical shape as a result of the fixation process, this shows the locations of the short lifetime regions in three dimensions. 3D reconstruction of the confocal z-stack images (see Figure S4) and quantitative analysis of the short lifetime regions were carried out using the Avizo software.

Table 4 shows the volume, mean lifetime, and position with respect to the radius of the nucleus of the short lifetime regions. The calculated volumes for the short lifetime regions are found to be larger than the typical volume of a metaphase chromosome (~1–3 um^3^), suggesting a considerable decompaction of chromatin in interphase. The positions of the short lifetime regions were calculated to determine if they have preferential locations in the nucleus. It can be observed from the table that the short lifetime regions are located closer to the periphery than the center of the nucleus. We examined three nuclei in this study and found that all showed a similar distribution of short lifetime regions (see Figures S6 and S7 for the other two nuclei).

**Figure 7:**
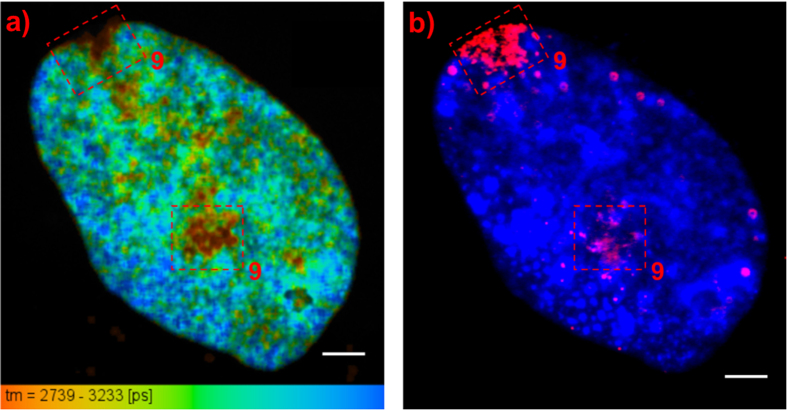
FLIM of interphase nucleus from CCD37LU cells. (**a**) Lifetime image of the nucleus. The short lifetime regions enclosed in a red dashed square were identified as part of chromosome 9 (scale bar = 5 μm). (**b**) FISH image of the measured nucleus showing the location of the centromere probe for chromosome 9 (scale bar = 5 μm). The location of the probe overlaps with that of the enclosed short lifetime regions in Fig. 7a.

DAPI-stained interphase chromosomes within a nucleus from CCD37LU lung fibroblast cells fixed in 3:1 methanol:acetic acid were also measured, as shown in Fig. 7a. The image shows the presence of short lifetime regions within the nucleus, similar to that observed in the GM18507 lymphocyte cells. However, the short lifetime regions have no preferential location within the nucleus. Figure 7b shows the FISH image of the measured nucleus. Chromosome 9 centromere probe is shown as red in the image. Comparing Fig. 7a,b, it can be observed that the location of the chromosome 9 centromere probe overlaps with that of the two of the short lifetime regions. Another measured nucleus shows a similar result and is presented in Figure S8.

## Discussion

We employed FLIM to study chromatin condensation across different human cell lines and cell cycle phases, finding that fluorescence lifetime is sensitive to variations in chromatin compaction. Variations in the lifetime of a commonly used fluorophore, DAPI, were observed along the length of metaphase chromosomes obtained from two cell lines: GM18507 lymphocyte and HeLa cells, where heteromorphic regions in the chromosomes were identified. Heteromorphic regions of chromosomes 1, 9, 15, 16, and Y displayed significantly shorter lifetime values than the rest of the chromosomes in the spread for the cell lines used signifying that the results are not cell line specific. Heteromorphic regions consist of highly condensed constitutive heterochromatin^27^, which is made up of satellite DNA, therefore suggesting that shorter DAPI lifetimes are associated with more condensed structures. This is supported by the strong correlation we have observed between DAPI lifetime and chromosome area, which represents the degree of chromatin condensation.

We suggest that differentially compacted chromatin leads to variation in DAPI lifetime due to supercoiling. More condensed chromatin structures are associated with stretched and positively supercoiled (overwound) DNA^1^,^28^,^29^,^30^. DNA stretching and overwinding causes the DNA minor groove width to decrease^29^ and therefore, DAPI molecules in more condensed regions have closer contact and interact more (e.g. through van der Waals and electrostatic forces) with the DNA sugar-phosphate backbone and base pairs in the minor groove. These intermolecular interactions can lead to the quenching of the DAPI fluorescence. To confirm our hypothesis that fluorescence lifetime variation in chromosomes is due to differentially compacted regions of chromatin, we undertook a series of experiments to discount other potential sources of lifetime variation.

First, we studied fluorescence lifetimes of DAPI free in solution and bound to two types of DNA with different GC content. In both cases, two lifetime components were observed, arising from fluorescence quenching via proton transfer to DAPI from the solvent^31^,^32^. In solution, most DAPI molecules are protonated and therefore quenched, yielding a short lifetime component, whilst the rest of the population is non-protonated and exhibits longer lifetimes. When bound to DNA, DAPI molecules bound externally or intercalating are less protected from solvent than those in the minor groove, again leading to populations with short and long lifetime components, respectively^31^,^33^. The lifetime of DAPI bound to human metaphase chromosomes exhibited one component. The single exponential decay of the DAPI fluorescence and the value of the lifetime signify that, at the concentration used in the experiments, DAPI molecules are protected from solvent quenching and are bound to the chromosomes mostly through minor groove binding to the DNA.

Next, we discounted the possibility of sequence specificity as the cause of lifetime variations by employing a concentration series. In AT-rich regions, there is a higher localised concentration of DAPI molecules and self-quenching caused by homo-energy transfer can occur, resulting in shorter lifetimes. When the concentration of DAPI was increased from 0.4 μM to 400 μM, the total signal intensity increased, implying an increase in the concentration of DAPI molecules bound to the chromosomes. There was no significant accompanying change in the DAPI lifetime. This suggests that there is no significant self-quenching of DAPI taking place at the concentration range used due to the lack of decrease in the DAPI lifetime. Hence, the concentration of DAPI in regions with differing AT content and thereby the sequence specificity of DAPI is not responsible for the reported lifetime variations. This is further supported by experiments on DAPI bound to DNA in solution wherein the AT content of the DNA did not significantly affect the lifetime of the minor groove bound DAPI molecules.

Since we have associated the lifetime variations with differences in chromatin compaction, we suggest that the shorter DAPI lifetimes observed for the heteromorphic regions of chromosomes 9 and 15 as compared with those of the other heteromorphic regions signify that these regions are more condensed than the other heteromorphic regions. The difference in the structures of the heteromorphic regions between different chromosomes may result from a difference in the type and amount of satellite DNA in these regions. The heteromorphic region of chromosome 9 is known to contain the bulk of the human satellite DNA^34^. Hence, the high concentration of satellite DNA in this region may lead to a more condensed structure, causing the very short lifetimes observed at this region as compared to those of the other heteromorphic regions. Furthermore, it was shown by Gosden *et al*.^34^ and Jones *et al*.^35^ that human satellite III DNA concentrates mostly at the heteromorphic regions of chromosomes 9 and 15 while human satellite II DNA concentrates mostly at the heteromorphic regions of chromosomes 1, 16, and Y. Satellite III displays more sequence divergence than satellite II^35^,^36^ and this difference may be one of the factors that lead to the difference in the structures, and therefore fluorescence lifetimes, of the heteromorphic regions.

Short lifetime regions were also observed for interphase nuclei from GM18507 lymphocyte and CCD37LU lung fibroblast cells. From our metaphase chromosome data, we suggest that these short lifetime regions consist of heterochromatin and may correspond to the heteromorphic regions observed for the metaphase chromosomes. This is further supported by the FISH images of lung fibroblast nuclei, as short lifetime regions overlap with a centromere probe targeting chromosome 9, one of the chromosomes containing the heteromorphic regions. Shorter DAPI lifetime, as compared with the rest of the chromosomes, was also observed at the heteromorphic region of chromosome 9 at metaphase for the CCD37LU cells (see Figure S9).

The calculated positions of the heterochromatic blocks inside the lymphocyte nucleus agree with several studies performed on the formation of chromosome territories inside an interphase nucleus^37^,^38^,^39^,^40^,^41^. For spherical interphase nuclei, the radial positioning of the chromosomes correlates with their gene density^37^,^38^. The late-replicating and gene-poor chromosomes, such as chromosomes 9 and Y, are preferentially located near the periphery of the nucleus. Even though chromosomes 1 and 16 are not preferentially located near the periphery of the nucleus due to their high gene density, their heterochromatic blocks, which are late-replicating and gene-poor, tend to position near the periphery^37^,^39^,^40^,^41^. No preferential location was observed for heterochromatic blocks inside lung fibroblast nuclei. Lung fibroblast nucleus is ellipsoidal unlike the spherical nucleus from lymphocyte cells. It is possible that the type and shape of the nucleus affects the positioning of the chromosomes.

Our FLIM results from metaphase and interphase chromosomes (see Figure S10) stained with Hoechst 33258 further show that the use of FLIM for chromosome structural investigation is not unique to DAPI. Since the observed lifetime variations in the Hoechst 33258-stained metaphase chromosomes were not as high as those observed for the DAPI-stained metaphase chromosomes, we suggest that DAPI molecules are more sensitive to changes in chromatin environment than Hoechst 33258. This could be due to the differences in their structures which may result in a different mechanism and rate for the quenching of their fluorescence. It is highly likely that other DNA binding probes may be more sensitive to the chromosome structure than DAPI.

In summary, we have shown in this study that FLIM can be effectively used to probe chromosome structure and map out locations of differentially compacted regions of chromatin along the chromosome length and within the nucleus. Whilst our studies were carried out on fixed cells, there is scope for similar live-cell studies with suitable fluorophores. Any such study would be invaluable in probing the dynamics of chromatin condensation. Other future studies could include the FLIM characterisation of heteromorphic variants, which would have applications in clinical diagnostics. Studies in the effect of epigenetic variations on structure could also benefit from FLIM.

## Additional Information

**How to cite this article**: Estandarte, A. K. *et al*. The use of DAPI fluorescence lifetime imaging for investigating chromatin condensation in human chromosomes. *Sci. Rep.* **6**, 31417; doi: 10.1038/srep31417 (2016).

## Supplementary Material

Supplementary Information

## Figures and Tables

**Table 1:** Lifetime values for free DAPI and DAPI bound to DNA.

	a_1_ ± SD	τ_1_ ± SD, ns	a_2_ ± SD	τ_2_ ± SD, ns
DAPI in Solution	0.60	0.36	0.40	2.66
ML DNA	0.40 ± 0.06	0.63 ± 0.04	0.60 ± 0.06	2.23 ± 0.02
CT DNA	0.42 ± 0.02	0.50 ± 0.08	0.58 ± 0.02	2.18 ± 0.06

**Table 2:** Lifetime values for the heteromorphic regions.

Chromosome #	Mean Lifetime ± SD, ns
1	2.58 ± 0.06
9a	2.38 ± 0.06
9b	2.21 ± 0.05
15	2.43 ± 0.05
16	2.55 ± 0.06
Y	2.58 ± 0.03

**Table 3:** Change in lifetime with DAPI concentration.

Concentration, μM	Mean Lifetime ± SD, ns	Intensity ± SD (x10^6^), arb. units
0.4	2.98 ± 0.01	7.63 ± 0.71
4	2.91 ± 0.04	9.75 ± 0.74
40	2.90 ± 0.03	10.7 ± 0.60
400	3.05 ± 0.02	14.1 ± 1.77

**Table 4:** Quantitative analysis of the short lifetime regions.

Region #	Volume, μm^3^	Distance*/Radius of Nucleus	Mean Lifetime ± SD, ns
1	8.25	0.90	2.57 ± 0.08
2	8.02	0.90	2.55 ± 0.06
3	0.16	0.77	2.44 ± 0.03
4	0.83	0.71	2.24 ± 0.05
5	0.13	0.68	2.45 ± 0.04
6	0.80	0.59	2.28 ± 0.03
7	1.90	0.54	2.56 ± 0.04
8	6.14	0.48	2.55 ± 0.05
9	5.22	0.47	2.54 ± 0.06

*The distance corresponds to that between the center of mass (COM) of the nucleus and the COM of the short lifetime regions.

## References

[b1] Richmond T. J. & Davey C. A. The structure of DNA in the nucleosome core. Nature 423, 145–150 (2003). 12736678 10.1038/nature01595

[b2] Finch J. & Klug A. Solenoidal model for superstructure in chromatin. Proc. Natl. Acad. Sci. USA. 73, 1897–1901 (1976). 1064861 10.1073/pnas.73.6.1897 PMC430414

[b3] Laemmli U. . In Cold Spring Harb. Symp. Quant. Biol. Vol. 42, 351–360 (Cold Spring Harbor Laboratory Press, 1978). 277351 10.1101/sqb.1978.042.01.036

[b4] Heitz E. Das Heterochromatin der Moose, 1. Jahrb. Wiss. Bot. 69, 762–818 (1928).

[b5] Craig J. M. & Bickmore W. A. Genes and genomes: Chromosome bands–flavours to savour. Bioessays 15, 349–354 (1993). 8343145 10.1002/bies.950150510

[b6] Comings D. E. Mechanisms of chromosome banding and implications for chromosome structure. Annu. Rev. Genet. 12, 25–46 (1978). 85431 10.1146/annurev.ge.12.120178.000325

[b7] Dillon N. & Festenstein R. Unravelling heterochromatin: competition between positive and negative factors regulates accessibility. Trends Genet. 18, 252–258 (2002). 12047950 10.1016/s0168-9525(02)02648-3

[b8] Trojer P. & Reinberg D. Facultative heterochromatin: is there a distinctive molecular signature? Mol. Cell 28, 1–13 (2007). 17936700 10.1016/j.molcel.2007.09.011

[b9] Jacobs P. A. & Klinger D. S. H. P. Paris conference (1971): Standardization in human cytogenetics. Cytogenet. Genome Res. 11, 313–362 (1972). 4647417

[b10] Periasamy A. & Clegg R. FLIM Microscopy in Biology and Medicine, Ch. 2, 35–59 (Taylor & Francis Group, LLC, 2010).

[b11] Gadella T. & Jovin T. M. Oligomerization of epidermal growth factor receptors on A431 cells studied by time-resolved fluorescence imaging microscopy. A stereochemical model for tyrosine kinase receptor activation. J. Cell Biol. 129, 1543–1558 (1995). 7790353 10.1083/jcb.129.6.1543 PMC2291181

[b12] Bastiaens P. & Jovin T. M. Microspectroscopic imaging tracks the intracellular processing of a signal transduction protein: fluorescent-labeled protein kinase C beta I. Proc. Natl. Acad. Sci. USA 93, 8407–8412 (1996). 8710884 10.1073/pnas.93.16.8407 PMC38684

[b13] Botchway S. W., Parker A. W., Bisby R. H. & Crisostomo A. G. Real‐time cellular uptake of serotonin using fluorescence lifetime imaging with two‐photon excitation. Microsc. Res. Tech. 71, 267–273 (2008). 18080329 10.1002/jemt.20548

[b14] Hamann S. . Measurement of cell volume changes by fluorescence self-quenching. J. Fluoresc. 12, 139–145 (2002).

[b15] Llères D., James J., Swift S., Norman D. G. & Lamond A. I. Quantitative analysis of chromatin compaction in living cells using FLIM–FRET. J. Cell Biol. 187, 481–496 (2009). 19948497 10.1083/jcb.200907029 PMC2779238

[b16] Kapuscinski J. DAPI: a DNA-specific fluorescent probe. Biotech. Histochem. 70, 220–233 (1995). 8580206 10.3109/10520299509108199

[b17] Manzini G., Barcellona M., Avitabile M. & Quadrifoglio F. Interaction of diamidino-2-phenylindole (DAPI) with natural and synthetic nucleic acids. Nucleic Acids Res. 11, 8861–8876 (1983). 6672773 10.1093/nar/11.24.8861 PMC326630

[b18] Kubista M., Aakerman B. & Norden B. Characterization of interaction between DNA and 4′, 6-diamidino-2-phenylindole by optical spectroscopy. Biochemistry 26, 4545–4553 (1987). 3663606 10.1021/bi00388a057

[b19] Barcellona M. . DNA-4′-6-diamidine-2-phenylindole interactions: A comparative study employing fluorescence and ultraviolet spectroscopy. Arch. Biochem. Biophys. 250, 48–53 (1986). 3767381 10.1016/0003-9861(86)90700-9

[b20] Cavatorta P., Masotti L. & Szabo A. A time-resolved fluorescence study of 4′, 6′-diamidine-2-phenylindole dihydrochloride binding to polynucleotides. Biophys. Chem. 22, 11–16 (1985). 17007778 10.1016/0301-4622(85)80021-1

[b21] Wilson W. D. . Binding of 4′, 6-diamidino-2-phenylindole (DAPI) to GC and mixed sequences in DNA: intercalation of a classical groove-binding molecule. J. Am. Chem. Soc. 111, 5008–5010 (1989).

[b22] Trotta E., D’Ambrosio E., Ravagnan G. & Paci M. Evidence for DAPI intercalation in CG sites of DNA oligomer [d (CGACGTCG)] 2: a 1H NMR study. Nucleic Acids Res. 23, 1333–1340 (1995). 7753623 10.1093/nar/23.8.1333 PMC306858

[b23] Shemilt L., Estandarte A., Yusuf M. & Robinson I. Scanning electron microscope studies of human metaphase chromosomes. Phil. Trans. R. Soc. A. 372, 20130144 (2014). 24470422 10.1098/rsta.2013.0144 PMC3900039

[b24] Yusuf M., Leung K., Morris K. J. & Volpi E. V. Comprehensive cytogenomic profile of the *in vitro* neuronal model SH-SY5Y. Neurogenetics 14, 63–70 (2013). 23224213 10.1007/s10048-012-0350-9 PMC3569589

[b25] Botchway S. W. . A series of flexible design adaptations to the Nikon E‐C1 and E‐C2 confocal microscope systems for UV, multiphoton and FLIM imaging. J. Microsc. 258, 68–78 (2015). 25664385 10.1111/jmi.12218

[b26] Barcellona M. & Gratton E. A molecular approach to 4′, 6-diamidine-2-phenylindole (DAPI) photophysical behaviour at different pH values. Biophys. Chem. 40, 223–229 (1991). 1655085 10.1016/0301-4622(91)80022-j

[b27] Wyandt H. E. & Tonk V. S. Atlas of Human Chromosome Heteromorphisms, Ch. 1, 17–22 (Springer, 2003).

[b28] Naughton C. . Transcription forms and remodels supercoiling domains unfolding large-scale chromatin structures. Nat. Struct. Mol. Biol. 20, 387–395 (2013). 23416946 10.1038/nsmb.2509 PMC3689368

[b29] Suto R. K. . Crystal structures of nucleosome core particles in complex with minor groove DNA-binding ligands. J. Mol. Biol. 326, 371–380 (2003). 12559907 10.1016/s0022-2836(02)01407-9

[b30] Schalch T., Duda S., Sargent D. F. & Richmond T. J. X-ray structure of a tetranucleosome and its implications for the chromatin fibre. Nature 436, 138–141 (2005). 16001076 10.1038/nature03686

[b31] Barcellona M. & Gratton E. The fluorescence properties of a DNA probe. Eur. Biophys. J. 17, 315–323 (1990). 2307139 10.1007/BF00258380

[b32] Mazzini A., Cavatorta P., Iori M., Favilla R. & Sartor G. The binding of 4′, 6-diamidino-2-phenylindole to bovine serum albumin. Biophys. Chem. 42, 101–109 (1992). 1581510 10.1016/0301-4622(92)80012-t

[b33] Barcellona M. L. & Gratton E. Fluorescence lifetime distributions of DNA-4′, 6-diamidino-2-phenylindole complex. Biochim. Biophys. Acta 993, 174–178 (1989). 2597690 10.1016/0304-4165(89)90160-8

[b34] Gosden J., Mitchell A., Buckland R., Clayton R. & Evans H. The location of four human satellite DNAs on human chromosomes. Exp. Cell Res. 92, 148–158 (1975). 48464 10.1016/0014-4827(75)90648-5

[b35] Jones K., Prosser J., Corneo G. & Ginelli E. The chromosomal location of human satellite DNA III. Chromosoma 42, 445–451 (1973). 4730561 10.1007/BF00399411

[b36] Corneo G., Ginelli E. & Polli E. Renaturation properties and localization in heterochromatin of human satellite DNA’s. Biochim. Biophys. Acta 247, 528–534 (1971). 5141664 10.1016/0005-2787(71)90689-7

[b37] Boyle S. . The spatial organization of human chromosomes within the nuclei of normal and emerin-mutant cells. Hum. Mol. Genet. 10, 211–219 (2001). 11159939 10.1093/hmg/10.3.211

[b38] Tanabe H., Habermann F. A., Solovei I., Cremer M. & Cremer T. Non-random radial arrangements of interphase chromosome territories: evolutionary considerations and functional implications. Mutat. Res. 504, 37–45 (2002). 12106644 10.1016/s0027-5107(02)00077-5

[b39] Ferreira J., Paolella G., Ramos C. & Lamond A. I. Spatial organization of large-scale chromatin domains in the nucleus: a magnified view of single chromosome territories. J. Cell Biol. 139, 1597–1610 (1997). 9412456 10.1083/jcb.139.7.1597 PMC2132633

[b40] Sadoni N. . Nuclear organization of mammalian genomes Polar chromosome territories build up functionally distinct higher order compartments. J. Cell Biol. 146, 1211–1226 (1999). 10491386 10.1083/jcb.146.6.1211 PMC2156120

[b41] Cremer T. & Cremer C. Chromosome territories, nuclear architecture and gene regulation in mammalian cells. Nat. Rev. Genet. 2, 292–301 (2001). 11283701 10.1038/35066075

