# Supplementary material for: The use of DAPI fluorescence lifetime imaging for investigating chromatin condensation in human chromosomes
Source: Sci Rep. 2016 Aug 16;6:31417. doi: 10.1038/srep31417 (PMC4985626; doi:10.1038/srep31417)
Supplement: Supplementary Information [file srep31417-s1.pdf]

The use of DAPI fluorescence lifetime imaging for investigating chromatin condensation in human chromosomes

Ana Katrina Estandarte,[1,2,*] Stanley Botchway,[3,*] Christophe Lynch,[1,2] Mohammed Yusuf,[1,2] and Ian Robinson[1,2,*]

[1] London Centre for Nanotechnology, University College London, London, WC1H 0AH, UK

[2] Research Complex at Harwell, Rutherford Appleton Laboratory, Oxon, OX11 0FA, UK

[3] Central Laser Facility, Science and Technology Facilities Council, Rutherford Appleton Laboratory, Oxon, OX11 0QX, UK

* To whom correspondence should be addressed. Tel: +44(0)1235567847; Fax: +44(0)1235567799; Email: ana.estandarte.11@ucl.ac.uk, stan.botchway@stfc.ac.uk, i.robinson@ucl.ac.uk

Supplementary Materials

Tables S1-S11. DAPI lifetime values for the heteromorphic regions of the other measured metaphase chromosome spreads.

Slide 1

Table S1. Chromosome spread 4_395_c

Chromosome #	Mean Lifetime ± SD, ns
1	2.56 ± 0.06
9a	2.46 ± 0.06
9b	2.25 ± 0.05
15	2.44 ± 0.03
16	2.57 ± 0.06
Y	2.59 ± 0.05

Table S2. Chromosome spread 4_318_a

Chromosome #	Mean Lifetime ± SD, ns
1	2.42 ± 0.06
9a	2.31 ± 0.06
9b	2.15 ± 0.04
15	2.33 ± 0.02
16	2.42 ± 0.05
Y	2.44 ± 0.03

Table S3. Chromosome spread 4_410_b

Chromosome #	Mean Lifetime ± SD, ns
1	2.64 ± 0.06
9a	2.54 ± 0.05
9b	2.37 ± 0.04
15	2.51 ± 0.04
16	2.63 ± 0.05
Y	2.66 ± 0.05

Slide 2

Table S4. Chromosome spread 4_71_a

Chromosome #	Mean Lifetime ± SD, ns
1	2.55 ± 0.04
9a	2.47 ± 0.04
9b	2.33 ± 0.04
15	2.45 ± 0.05
16	2.55 ± 0.04
Y	2.56 ± 0.04

Table S5. Chromosome spread 4_139_e

Chromosome #	Mean Lifetime ± SD, ns
1	2.35 ± 0.04
9a	2.22 ± 0.05
9b	2.08 ± 0.04
15	2.25 ± 0.03
16	2.35 ± 0.04
Y	2.35 ± 0.05

Table S6. Chromosome spread 4_644_c

Chromosome #	Mean Lifetime ± SD, ns
1	2.54 ± 0.05
9a	2.23 ± 0.04
9b	2.11 ± 0.04
15	2.39 ± 0.03
16	2.55 ± 0.05
Y	2.55 ± 0.04

Table S7. Chromosome spread 4_411_c

Chromosome #	Mean Lifetime ± SD, ns
1	2.71 ± 0.04
9a	2.61 ± 0.05
9b	2.47 ± 0.04
15	2.57 ± 0.04
16	2.72 ± 0.05
Y	2.74 ± 0.05

Slide 3

Table S8. Chromosome spread 5_168_a

Chromosome #	Mean Lifetime ± SD, ns
1	2.24 ± 0.05
9a	2.23 ± 0.04
9b	2.09 ± 0.04
15	2.11 ± 0.04
16	2.25 ± 0.05
Y	2.24 ± 0.05

Table S9. Chromosome spread 5_149_a

Chromosome #	Mean Lifetime ± SD, ns
1	2.31 ± 0.05
9a	2.31 ± 0.05
9b	2.17 ± 0.02
15	2.21 ± 0.01
16	2.32 ± 0.05
Y	2.33 ± 0.03

Table S10. Chromosome spread 5_555_a

Chromosome #	Mean Lifetime ± SD, ns
1	2.29 ± 0.05
9a	2.29 ± 0.04
9b	2.15 ± 0.03
15	2.16 ± 0.05
16	2.28 ± 0.05
Y	2.33 ± 0.05

Table S11. Chromosome spread 5_872_b

Chromosome #	Mean Lifetime ± SD, ns
1	2.30 ± 0.04
9a	2.29 ± 0.03
9b	2.20 ± 0.04
15	2.20 ± 0.03
16	2.31 ± 0.05
Y	2.29 ± 0.04

Table S12. Lifetime values for the Hoechst 33258-stained chromosomes containing the heteromorphic regions.

Chromosome #	Mean Lifetime ± SD for Heteromorphic Region, ns	Mean Lifetime ± SD for Rest of the Chromosome, ns
1	2.35 ± 0.03	2.43 ± 0.04
9	2.35 ± 0.02	2.44 ± 0.03
15	2.34 ± 0.01	2.44 ± 0.03
16	2.36 ± 0.01	2.42 ± 0.03
Y	2.38 ± 0.02	2.47 ± 0.03

* The mean lifetimes and standard deviations obtained for chromosomes with the same chromosome number were averaged and pooled, respectively.

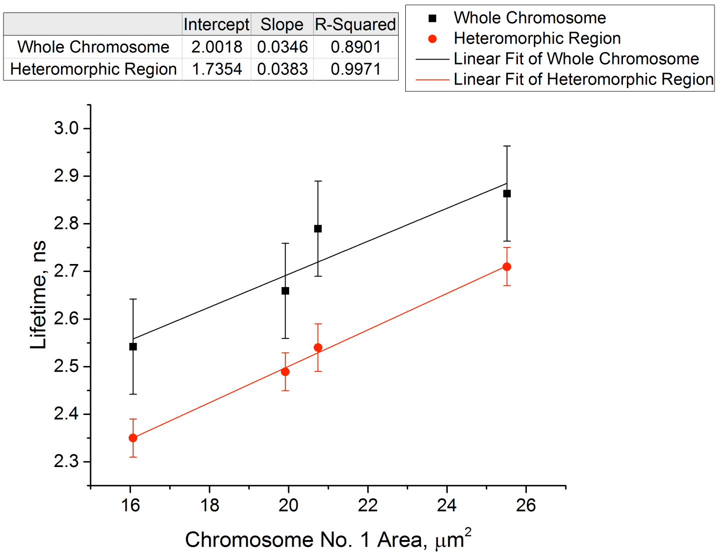

Figure S1. Mean fluorescence lifetime of DAPI for various chromosome 1's on another measured slide and their heteromorphic regions plotted against the area of the chromosomes. The error bars represent the standard deviation.

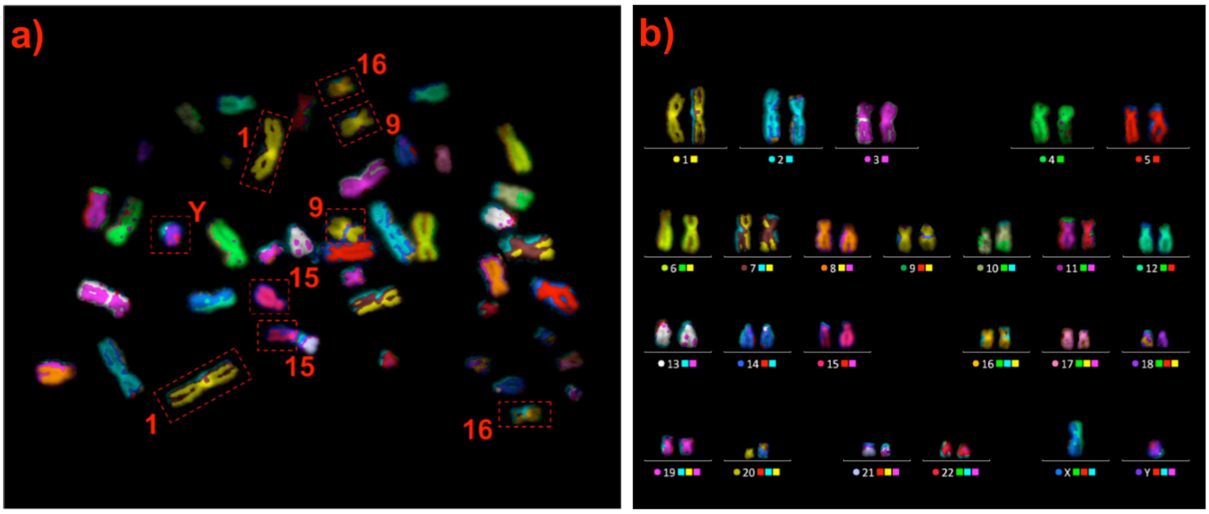

Figure S2. Identification of the chromosomes stained with Hoechst 33258. (*a*) mFISH image of the chromosome spread. (*b*) Karyotyping of the chromosomes in Figure S2*a* based on color.

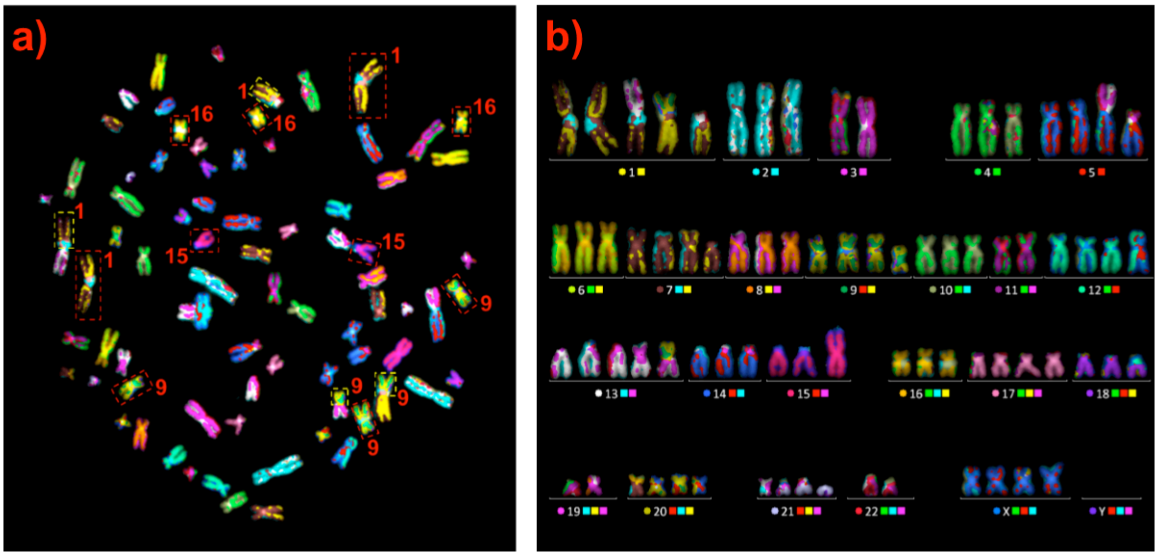

Figure S3. Identification of the chromosomes from HeLa cell line. (*a*) mFISH image of the chromosome spread. Chromosomes enclosed in a yellow dashed square are the abnormal chromosomes consisting a part of either chromosome 1 or 9. (*b*) Karyotyping of the chromosomes in Figure S3*a* based on color.

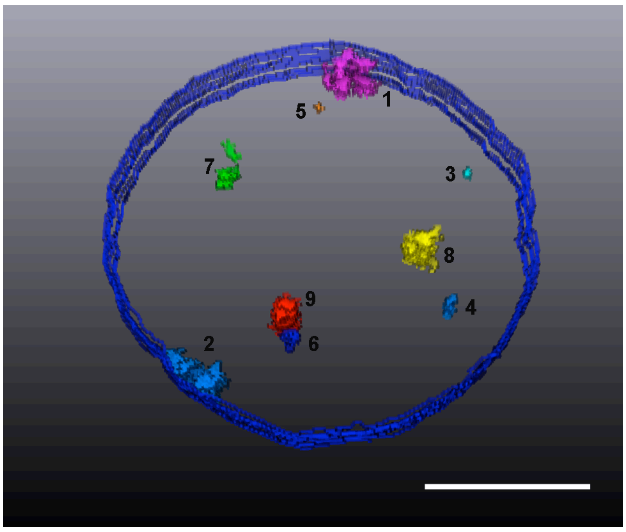

Figure S4. 3D stack of the lifetime images of a nucleus in Figure S5 showing the positions of the short lifetime regions inside the nucleus (scale bar = 10 μm).

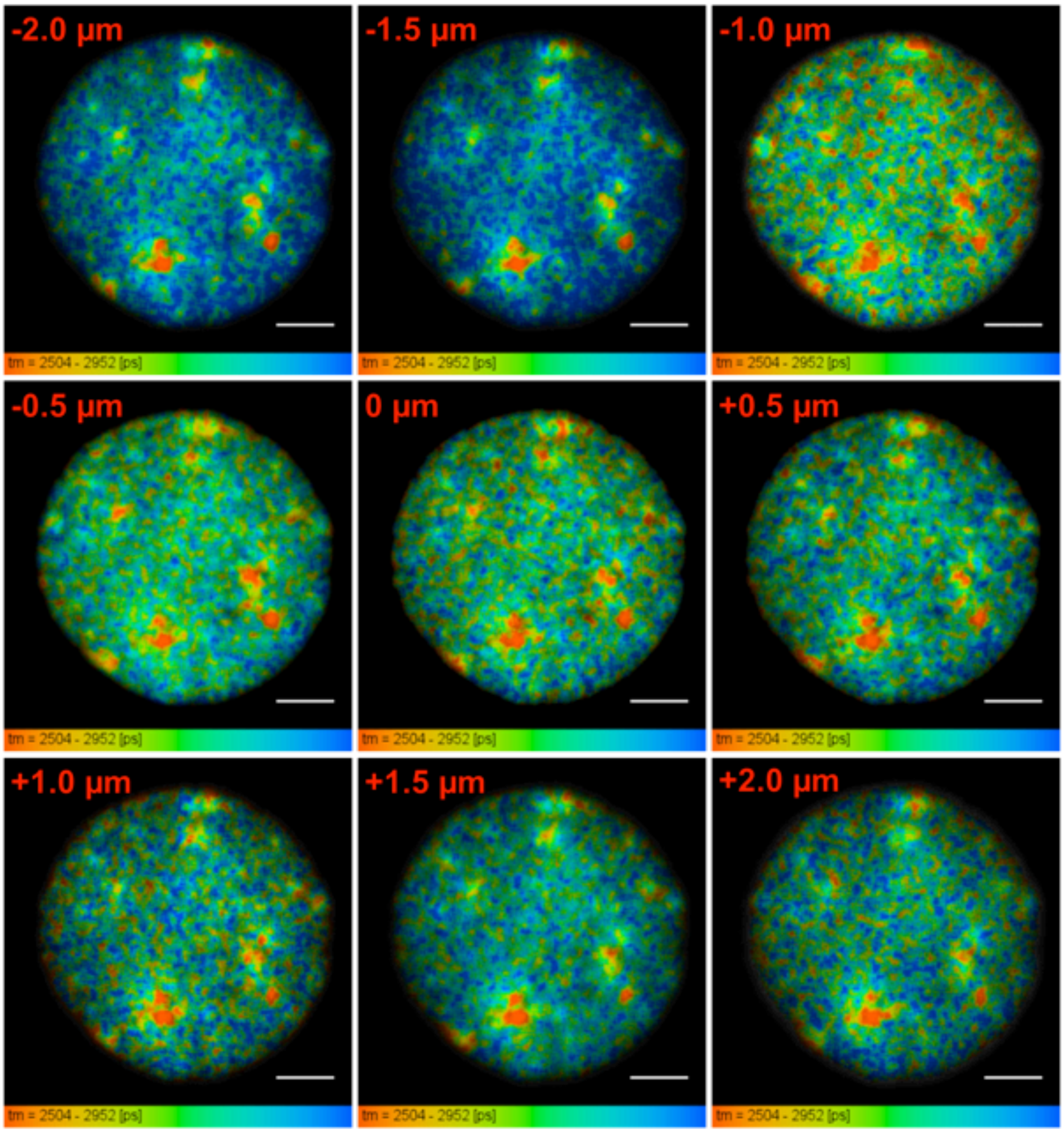

Figure S5. Z-stack of the lifetime images of a nucleus. Images are taken from -2.0 µm to +2.0 µm focus with 0.50-µm step sizes between the focal planes (scale bars = 5 µm).

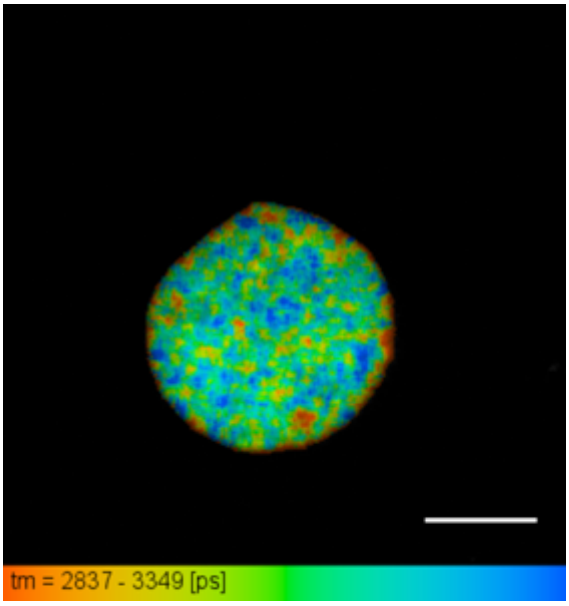

Figure S6. Lifetime image of another measured interphase nucleus showing regions of short lifetime values (scale bar = 10 µm).

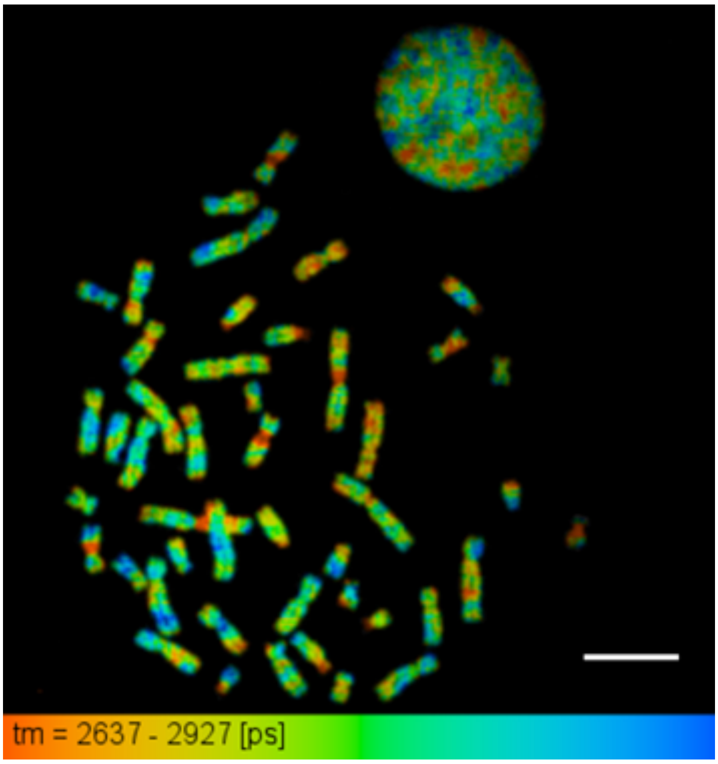

Figure S7. Lifetime image of a metaphase chromosome spread and an interphase nucleus showing regions of short lifetime values (scale bar = 10 µm).

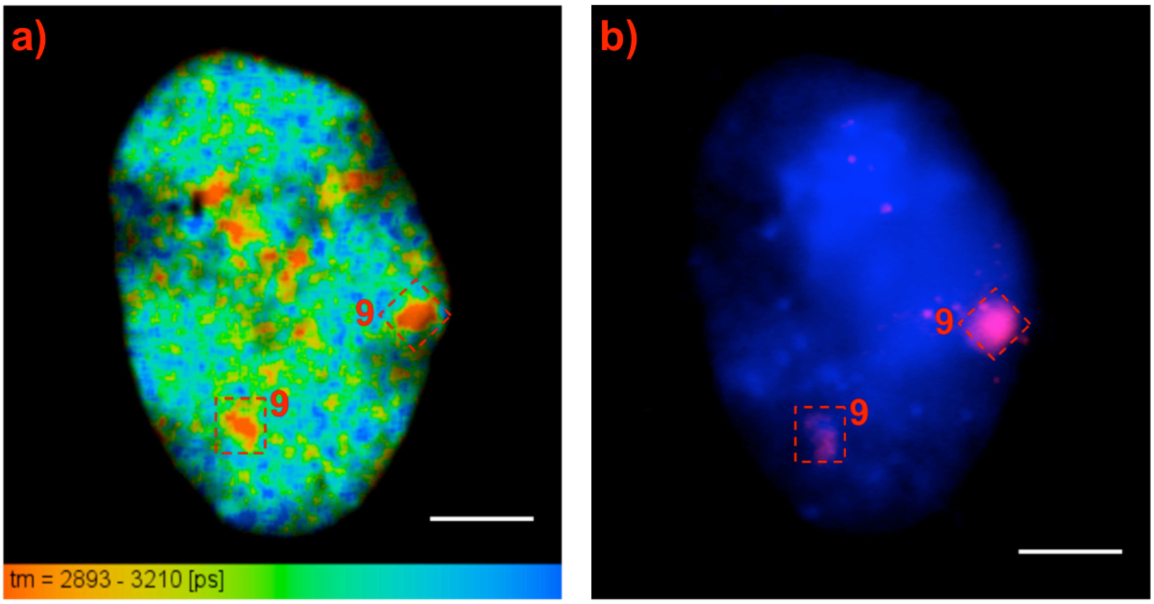

Figure S8. FLIM of another interphase nucleus from CCD37LU cells. (a) Lifetime image of the nucleus. The short lifetime regions enclosed in a red dashed square were identified as part of chromosome 9 (scale bar = 5 µm). (b) FISH image of the measured nucleus showing the location of the centromere probe for chromosome 9 (scale bar = 5 µm). The location of the probe overlaps with that of the enclosed short lifetime regions in Figure S8*a*.

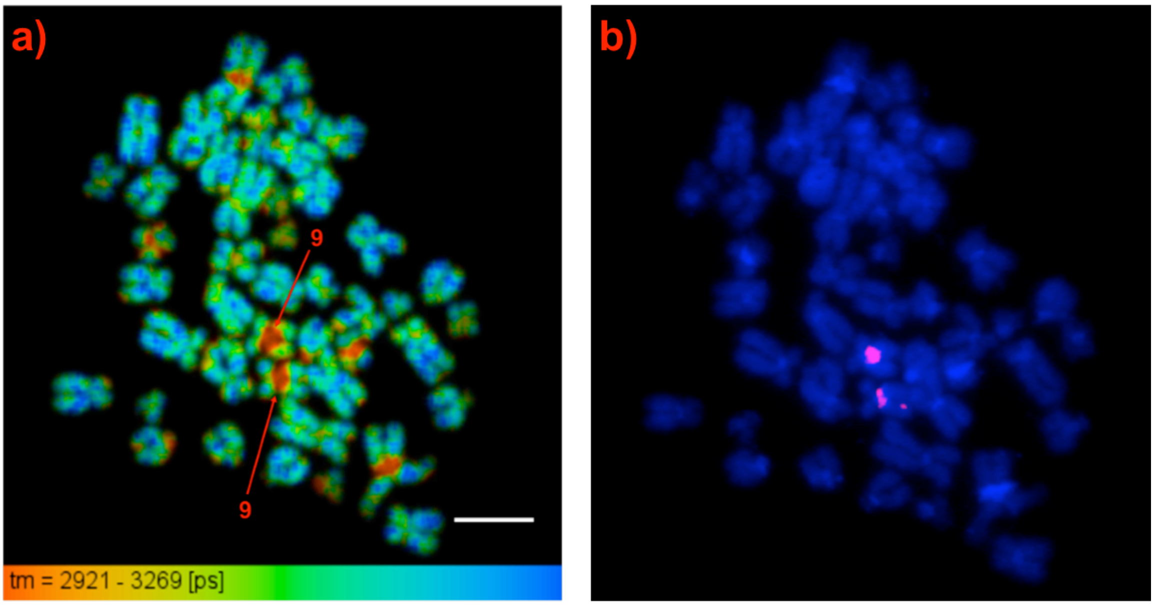

Figure S9. FLIM of metaphase chromosomes from CCD37LU cells. (*a*) Lifetime image of the measured spread (scale bar = 5 µm). Short lifetime regions (red regions) are observed along the length of some chromosomes. (*b*) FISH image of the spread showing the centromere probe for chromosome 9 in red. Two of the short lifetime regions in FigureS9*a* correspond to the heteromorphic region of chromosome 9.

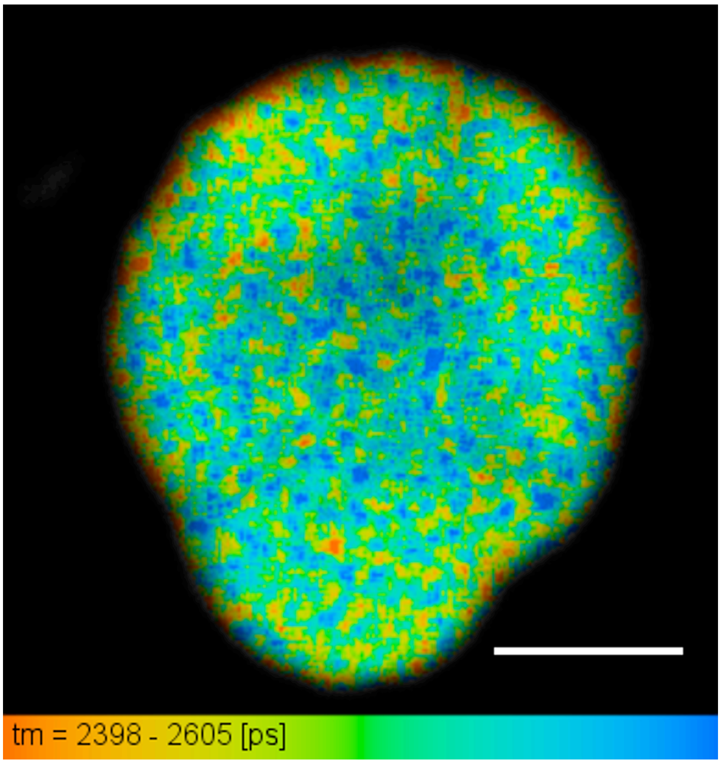

Figure S10. FLIM of Hoechst 33258-stained interphase nucleus from GM18507 cells (scale bar = 4 μm). Short lifetime regions are observed near the periphery of the nucleus.
